# Viral Interference and Persistence in Mosquito-Borne Flaviviruses

**DOI:** 10.1155/2015/873404

**Published:** 2015-10-25

**Authors:** Juan Santiago Salas-Benito, Mónica De Nova-Ocampo

**Affiliations:** ^1^Programa Institucional de Biomedicina Molecular, Escuela Nacional de Medicina y Homeopatía, Instituto Politécnico Nacional, 07320 Mexico, DF, Mexico; ^2^Programa de Doctorado en Ciencias en Biotecnología, Instituto Politécnico Nacional, 07320 Mexico, DF, Mexico

## Abstract

Mosquito-borne flaviviruses are important pathogens for humans, and the detection of two or more flaviviruses cocirculating in the same geographic area has often been reported. However, the epidemiological impact remains to be determined. Mosquito-borne flaviviruses are primarily transmitted through *Aedes* and *Culex* mosquitoes; these viruses establish a life-long or persistent infection without apparent pathological effects. This establishment requires a balance between virus replication and the antiviral host response. Viral interference is a phenomenon whereby one virus inhibits the replication of other viruses, and this condition is frequently associated with persistent infections. Viral interference and persistent infection are determined by several factors, such as defective interfering particles, competition for cellular factors required for translation/replication, and the host antiviral response. The interaction between two flaviviruses typically results in viral interference, indicating that these viruses share common features during the replicative cycle in the vector. The potential mechanisms involved in these processes are reviewed here.

## 1. Introduction

The Flaviviridae family includes approximately 80 members divided into four genera:* Flavivirus*,* Pestivirus*,* Pegivirus*, and* Hepacivirus*. The* Flavivirus* genus can be further divided into four categories: mosquito-borne, tick-borne, no known vector (NKV), and insect-specific (ISF) viruses [1]. Mosquito- and tick-borne flaviviruses, including Dengue (with four serotypes), Japanese encephalitis virus (JEV), Yellow fever virus (YFV), Saint Louis encephalitis virus (SLEV), West Nile virus (WNV), Murray Valley encephalitis virus (MVE), and tick-borne encephalitis virus (TBEV) [2], are important pathogens responsible for human diseases, such as encephalitis, fever, and haemorrhagic fever.

NKV viruses are primarily restricted to bats and rodents. ISF viruses are restricted to mosquitoes, such as* Culex* and* Aedes* [1], and include the* Aedes* flavivirus (AeFV), Calbertado virus, cell-fusing agent virus (CFAV), Chaoyang virus,* Culex* flavivirus (CxFV),* Culex theileri* flavivirus (CTFV), Kamiti River virus (KRV), Lammi virus, Nakiwogo virus (NAKV), Nounane virus, Quang Binh virus (QBV), and Palm Creek virus (PCV) [3–12].

Mosquito- and tick-borne flaviviruses are transmitted to humans through haematophagous insects during blood meal feeding. Viruses obtained from vertebrate host initially replicate in the midgut within 5–7 minutes of exposure. After escaping the midgut, the virus spreads to other tissues via haemolymph and can be transmitted through infected salivary glands and saliva [13–15]. The period from the initial infection in the midgut to when the vector transmits the virus is termed the extrinsic incubation period (EIP), and this time period varies from 7 to 14 days [14, 15]. However, viruses have been detected in the salivary glands of DENV-infected* Aedes aegypti* as early as 24 hours [16]. Thus, the EIP depends on the virus, the mosquito, and certain environmental factors [14, 15].

Flaviviruses are single stranded-RNA viruses with positive polarity (ssRNA+). These viruses are approximately 11 kb in length, with a single open reading frame (ORF) encoding a polyprotein that is co- and posttranslationally processed through cellular and viral proteases into three structural (C, M, and E) and seven nonstructural (NS1, NS2A, NS2B, NS3, NS4A, NS4B, and NS5) proteins. The ORF is flanked at the 5′ and 3′ ends by two untranslated regions (5′ and 3′ UTRs) that are important in viral translation and replication [2].

The virions are spherical and approximately 50 nm in diameter. The capsid (C) protein interacts with the viral genome to form the nucleocapsid, which is surrounded by a lipid bilayer containing the membrane (M) and envelope (E) proteins [17].

## 2. Persistence

Interestingly, flavivirus infections in humans are self-limiting episodes with or without pathological consequences, despite a life-long or persistent infection in the vector, without apparent pathological effects [13, 18–20]. In contrast to acute infections, where the virus is eventually eliminated through the destruction of the host or the immune response, in persistent infections, the virus remains in the host cells for long periods of time, can be transmitted to other cells, and is not cleared. Persistent infections facilitate viral transmission to vertebrate hosts [21].

Mosquito cell cultures have been used as suitable models to study flavivirus persistence (Table 1) because these models are easy to manage, they have several variables and can be controlled, and their results are typically easy to interpret.

One of the common features observed in C6/36 persistently infected with DENV [22–24] is the presence of a cytopathic effect (CPE) during the first 4–7 weeks of infection, which becomes progressively imperceptible throughout the infection [23, 24]. However, the viral proteins and genome are clearly detected in infected cells [22, 25]. These findings are consistent with the nonpathogenic flavivirus infection in mosquitoes, suggesting the presence of a balance between virus replication and the antiviral host response [26, 27], which results in an infection that is controlled, but not eliminated, and not lethal [21, 28]. The mechanisms underlying the establishment and maintenance of persistent flaviviral infection in insects are not completely understood, but apparently both host and viral factors are involved [28–30]. For example, viral titres in the supernatants of C6/36 [22, 24, 31] or TRA-171 cells [32] persistently infected with DENV display an oscillation pattern during persistent infection, suggesting the presence of defective interfering particles (DIs), one of the major self-controlling mechanisms for viral replication [33]. Because DIs are more evident after several passages in cell lines, these particles are frequently observed in persistently infected cultures [34], and the oscillating phenomenon observed in long-term or persistently infected cell cultures reflects a mutual interaction between the parental virus, required for the replication of both defective and standard viruses, and the viral interference induced through DIs in the viral titres [34]. A more detailed description of DIs is included in a subsequent section of this review.

Among the host factors that might participate in the maintenance of persistent infection are those associated with cell antiviral mechanisms, particularly RNA interference (RNAi) and the innate immune responses that control but do not eliminate viral infections. These mechanisms will be discussed later.

## 3. Viral Interference

Viral interference is a phenomenon whereby the infection with one virus (primary) inhibits infection with other viruses (secondary). Superinfection exclusion occurs when the primary virus inhibits the infection of the secondary virus. Additionally, when both viruses reciprocally inhibit their infections, this is referred to as intrinsic interference [35, 36]. When both viruses belong to the same family, the interference is referred to as homologous viral interference [37–39]. Heterotypic interference is a variation of this type of interference, and this phenomenon is observed when the viruses involved have different serotypes but belong to the same species, that is, DENV serotypes [30]. Heterologous viral interference describes a negative interaction between viruses from different families [38]. In some cases, the infection with two different viruses does not result in viral interference, and both infectious agents can coexist in the same cells. This phenomenon is called viral accommodation [40]. Based on the time of infection, mixed infections can be classified as coinfections when the two viruses interact with the host at the same time or superinfections when one virus invades the host prior to the second virus [41].

### 3.1. Viral Interference in Mosquito Cell Lines

Because flaviviral infection persists for the life of the vector, the opportunities for competition or viral interference in the vector are higher than in humans, where the infection is only transient and is cleared through the immune system [42]. The evaluation of viral interference during flavivirus infection is relatively easy to detect in cell lines, and it has primarily been examined in mosquito C6/36 cells (from* Aedes albopictus*) [12, 23, 30, 31, 35, 39, 41–48], TRA-171 (from* Toxorhynchites amboinensis*) [32], Sf9 (from* Spodoptera frugiperda*) [49], C7-10, and U4.4 cells (from* Aedes albopictus*) [29, 35]. Homologous or heterotypic, but not heterologous, viral interference is frequently observed during superinfections (Table 2), and this condition is particularly evident in persistently infected cells [23, 31, 32, 44, 49].

However, some exceptions have been documented [32, 39, 41, 44]. For example, SINV inhibits DENV replication in C6/36 cells infected 1 hour prior to DENV-4 [41]. The same cells persistently infected with Aal DNV and reinfected with DENV-2 showed an important reduction in the severity and mortality of the DENV-2 infection compared with those of noninfected cells, and DENV-2 titres were lower than in naïve cells [39]. Interestingly, C6/36 cells persistently infected with three different viruses, including two flaviviruses, DENV-2 and JEV, result in a stable coinfection with the three viruses without apparent viral interference [44]. These discrepancies indicate that the interference might vary among different flaviviruses [41] and might be influenced through both the type of virus and the cell line used. It has been shown that the interval between the primary and secondary viral infections has an important effect on viral interference. The primary infection of C6/36 cells with either DENV-2 or DENV-4, followed by a secondary infection 1 or 6 hours later with the opposite virus at the same multiplicity of infection (MOI), showed a stronger reduction in the virus titres of the secondary virus when the second infection was performed 6 hours after the first infection [42]. It is likely that DENV-4 infection requires more than 1 hour to establish conditions in which this virus will not be affected by SINV.

Contradictory results have been reported regarding WNV and CxFV infections. Some studies have reported that CxFV-infected C6/36 cells were reinfected with WNV 48 hours later and display significantly reduced titres of the secondary virus at 108 hours postinfection, indicating the presence of homologous viral interference [47]. However, other studies have reported that when the same CxFV-infected cells were reinfected with WNV two days later, homologous viral interference was not observed [45]. These differences could reflect the time of the secondary infections, but more experiments will be necessary to clarify this point.

Flavivirus coinfection experiments have primarily been performed using C6/36 cells [30, 41–43, 46, 48, 50], and recently Aag2 cells have been used [51]; these infections typically result in homologous or heterologous viral interference (Table 3).

### 3.2. Viral Interference in Mosquitoes

Experiments in mosquitoes have revealed some similarities to the findings obtained using mosquito cell lines. For example, C6/36 cells infected with DENV display heterotypic viral interference [30, 31, 42, 43, 50, 51].* Aedes aegypti* mosquitoes orally infected with DENV-2 and DENV-3 simultaneously showed higher amounts of DENV-2 viral RNA than DENV-3 viral RNA [50], suggesting that the replicative advantage of DENV-2 observed in C6/36 also applies to the vector.* Aedes aegypti* mosquitoes orally coinfected with two clades of DENV-2 (NI-1 and NI-2B) showed a higher replicative index for the NI-2B clade than the NI-1 clade [51]. Homologous viral interference has been observed in* Aedes aegypti* mosquitoes infected with DENV and YFV [18] or MVE and DENV [52],* Culex tritaeniorhynchus* mosquitoes infected with JEV and MVE [19], and* Culex quinquefasciatus* mosquitoes infected with other flaviviruses, such as WNV and SLEV [36].

The absence of heterologous viral interference between DENV and CHIKV has been documented in C6/36 cells [31, 46], and apparently this phenomenon also occurs in mosquitoes.* Aedes albopictus* mosquitoes intrathoracically inoculated with DENV-1 and subsequently with CHIKV via the oral route at 7 or 13 days later showed the presence of the two viruses, without superinfection exclusion. Mosquitoes orally coinfected with both viruses showed the same results, and the viruses could also be detected in saliva, indicating the absence of heterologous viral interference [53]. However, studies have shown that* Aedes aegypti* mosquitoes orally fed DENV and CHIKV did not exhibit dual infection, either in the same pool or in individual mosquitoes, suggesting the presence of heterologous viral interference [54]. The discrepancy of these results requires further investigation but apparently could reflect the virus strain used in these studies [53]. Interestingly,* Aedes albopictus* mosquitoes coinfected with DENV-4 and SINV display a reduction in both the infection and population dissemination rates compared with mosquitoes infected with DENV-4 alone, even when the vector used exhibits a low SINV infection rate [41].

Although contradictory results have been reported for WNV and CxFV infections in C6/36 cells, the findings reported for these two groups in mosquitoes are consistent.* Culex quinquefasciatus* mosquitoes inoculated with CxFV via an intrathoracic route and fed a blood meal containing WNV seven days later (superinfection) do not display superinfection exclusion. When both viruses were simultaneously inoculated (coinfection), both viruses were detected in the same mosquito tissues through immunofluorescence, indicating a physical interaction between these infectious agents [45]. In another study,* Culex pipiens* mosquitoes persistently infected with CxFV and orally challenged with WNV displayed a reduction in the dissemination rates only during the early stages of the infection (7 days), but not during the late stages (14 days), with no effects on the transmission rates [47]. Although both are flaviviruses, the absence of homologous viral interference could reflect differences between these viruses. For example, although the mosquito RNAi response might represent an antiviral response, the similarity between both viral genomes is not sufficient to generate a “cross-reaction,” particularly because the RNAi response is based on a highly specific-sequence mechanism. The absence of competition for cellular factors could also be a contributing factor because the 5′ and 3′ UTRs of CxFV have some differences compared with WNV [45]. Moreover, the mechanism of transmission between both viruses could also contribute to the absence of viral interference, as WNV is transmitted to the vertebrate host through saliva, and CxFV is maintained among the vector population through vertical transmission [47].

Unfortunately, the relevance of the viral interference in naturally infected vectors remains unknown, as several studies have demonstrated the presence of flavivirus coinfections that typically compete in both cell lines and mosquitoes. For example,* Aedes albopictus* or* Aedes aegypti* mosquitoes are naturally coinfected with more than one DENV serotype [55–57]. Although several studies have been performed using pools of mosquitoes where the coinfection in the same individual is difficult to determine [55, 57], other studies have clearly established that one individual can be infected with both DENV serotypes [56].

However, other data are consistent with the laboratory results. For example, the absence of viral interference between CHIKV and DENV in cell lines has been confirmed through the evidence of dual CHIKV and DENV-1 viral infections in* Aedes aegypti* mosquitoes [58] and in a single* Aedes albopictus* mosquito [59]. Similarly, 40% of* Culex* mosquitoes collected in Chicago, IL (USA), were naturally coinfected with CxFV and WNV, suggesting that in this vector homologous viral interference does not occur between these two flaviviruses, which are not closely related [60].

The mechanisms involved in viral interference remain elusive, but the inhibition could occur at different levels of the viral replicative cycle, such as binding, entry, replication, and morphogenesis [19, 37]. However, some studies have implicated several factors, such as DIs and the RNAi response, in homologous viral interference, and the competition for cellular replication factors and the innate immune response for heterotypic viral interference [47].

## 4. Defective Interfering Particles

The heterotypic viral interference against DENV-3 observed in C6/36 cells with an acute DENV-1 infection is affected through treatments with puromycin (an inhibitor of the protein synthesis) but not actinomycin D (an inhibitor of the cellular transcription), suggesting that the viral interference is predominantly mediated through the virus instead of cellular factors [30]. The competitive success of one virus could reflect the appropriation of the host cellular machinery for replication, which is directly related to the density of the viral genomes in the infected cells [42]. This idea might explain why the heterotypic viral interference between two different DENV serotypes is stronger when the interval between the primary and secondary infections is longer (e.g., between 1 and 6 hours) and weaker in a coinfection compared with superinfection [42]. This idea might also explain why this type of viral interference is observed in persistently infected cells [31].

One possible factor responsible for homologous viral interference is DIs. These virions contain a partially deleted genome, encoding generally normal viral structural proteins, with enough genomic information for replication and incorporation into mature virions; however, these viruses cannot perform their own replication. Therefore, they require the assistance of a standard “helper virus” for this process [20]. Because the genome is shorter, DIs are apparently preferentially replicated; therefore, these viruses obtain the viral genome density necessary to specifically interfere with the replication of the parental virus [33–35]. Thus, DIs represent a major self-controlling mechanism for viral replication [33], and these particles have been implicated in the establishment and maintenance of persistent viral infections [25, 33].

**Figure 1:**
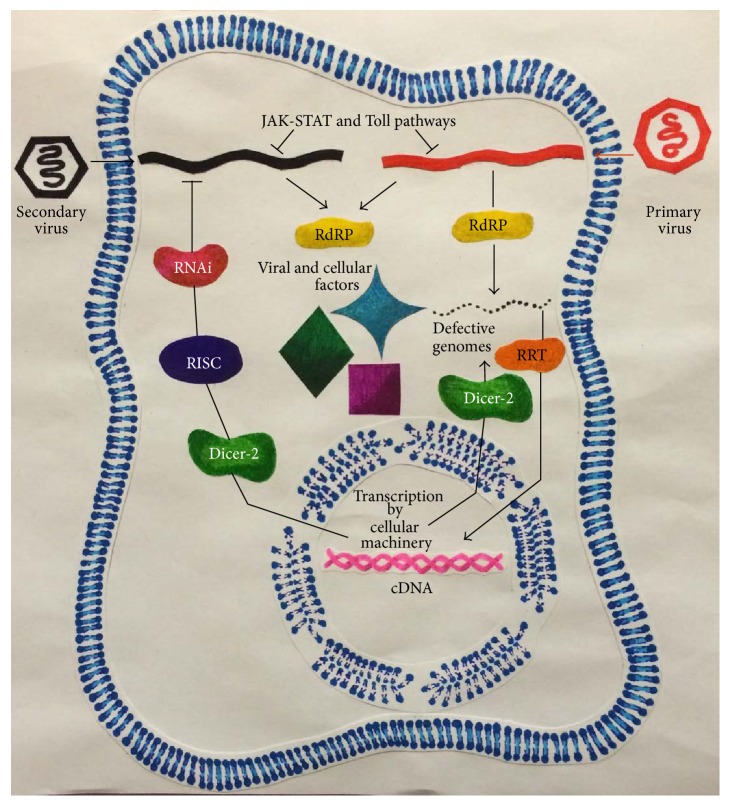
Mechanisms involved in viral interference/persistence. The JAK-STAT and Toll pathways represent the innate immune system. Viral RNA-dependent RNA polymerase (RdRP) and cellular Retrotransposon Retrotranscriptase (RRT) through Dicer-2 are involved in the generation of viral defective genomes which are able to compete for viral and cellular factors required for translation and/or replication of the parental virus. The viral cDNAs generated by the RRT could inhibit viral replication through Dicer-2 and the RNAi pathway. RISC, RNA-induced silencing complex.

The generation of DIs is a common feature among viruses and has been observed in both RNA and DNA viruses [20, 33]. The most commonly accepted mechanism for the generation of DIs is the participation of the viral polymerase, particularly in RNA-dependent viruses that lack “proofreading” activity. More recently, it has been proposed that* Drosophila melanogaster* cells persistently infected with several nonflavivirus RNA viruses generated cDNAs from the genomes of defective interfering particles through cellular retrotransposon reverse transcriptase-mediated retrotranscription (Figure 1), but this finding requires further investigation [28].

Defective viral genomes have been detected in mosquito cells persistently infected with flaviviruses, such as SLE [61], JEV [25], and DENV [24], and also those that occur in nature, circulating between mosquitoes and human populations [62]. These defective genomes have been implicated as cofactors in reducing the prevalence of DENV [62] and the severity of the disease in specific geographic areas [63].

In addition to the participation of defective interfering particles/genomes in viral interference, some mutations have recently been associated with this phenomenon.* Culex quinquefasciatus* mosquitoes orally superinfected with WNV containing a mutation in the 2K peptide (V9M) overcome the homologous viral interference typically observed in superinfections. The 2K peptide is a 23-amino-acid peptide located between the NS4A and NS4B proteins, and this peptide is anchored to the membrane of the endoplasmic reticulum (ER). The 2K peptide has been implicated in virus replication and the evasion of the RNase L-mediated antiviral response. This point mutation likely confers replication advantages, protection against RNA degradation, and/or the ability to compete with wild-type viruses under some circumstances [64].

## 5. Cellular Factors

Recent transcriptomic studies in* Aedes aegypti* mosquitoes infected with DENV-2 [16, 65–69] and* Culex pipiens quinquefasciatus* infected with WNV [70] have shown that these flaviviruses induce important and complex changes in gene expression. Although these studies have been performed with DENV and WNV, some preliminary experiments suggest that the expression of several genes is commonly activated through mosquito-borne flaviviruses [71, 72].

Although different results have been obtained, several studies have suggested that during flaviviral infection genes associated with specific pathways are activated to maintain the proper condition of the cell for viral replication, such as metabolism (nucleotide, lipid, amino acid, and energy) [65–68], oxidative stress [67, 68] and transcription/translation [65–68], and other pathways associated with the antiviral response [16, 65, 67, 68, 73]. Actually, some genes have been associated with the susceptibility or refractoriness to DENV infection in* Aedes* mosquitoes. For example, genes associated with the inhibition of the apoptosis were identified in susceptible [65] and proapoptotic genes in refractory mosquitoes [69]. Apoptosis is an antiviral mechanism used by the cell to eliminate viruses. Some genes associated with the immune response are upregulated in refractory mosquito strains [68, 73].

The requirement for cellular factors for viral replication could represent a homologous/heterotypic viral interference mechanism. The primary virus might sequester the host factors essential for the replication of the secondary virus [35, 37], which might result in viral interference, particularly during a superinfection (Figure 1). The absence of heterologous viral interference among nonrelated viruses might reflect the requirements for different cellular and viral factors to complete the viral replicative cycles. For example, DENV-2 is an RNA virus that assembles in the cytoplasm, while Aal DNV is a DNA virus that assembles in the nucleus [23]. When the viruses involved in the infection are closely related, the requirements for host factors are the same and the viral interference is stronger. Accordingly, C6/36 cells with a primary NHUV infection and a superinfection with WNV, SLEV, or JEV displayed a significant reduction in the secondary virus titre (homologous interference through superinfection). An analysis of the secondary structure of the NHUV 3′ UTR revealed similarities with the YFV and JEV serogroups and viruses in the tick-borne flavivirus clade [48], suggesting that the requirements for cellular factors and the mechanism for viral translation/replication might be similar among these viruses, thereby establishing conditions for competition. In Aag2 cells coinfected with two DENV-2 clades from Nicaragua, clade NI-2B showed a replicative preference over clade NI-1. Both viruses exhibited differences in some amino acids in two structural and four nonstructural proteins and showed four nucleotide variations in the 5′ and 3′ UTRs. These changes could influence some steps of the viral replicative cycle, resulting in advantages of one virus over the other [51]. Interestingly, although Aag2 cells are persistently infected with CFAV [74], the DENV infection is not affected, probably by the same cause that CxFV does not interfere with WNV replication in mosquitoes [45, 47].

Because members of the* Flavivirus* genus have ssRNA+, these viruses use a common strategy similar to other RNA viruses to translate the genome (revised in [75]). These viruses release viral RNA into the cytoplasm for recognition by both the viral replication apparatus and the translational cell machinery to assemble the RNA replication complex on cellular membranes [76–78].

Viral translation and replication cannot occur at the same time because the ribosome moves from the 5′ end towards the 3′ end of the RNA to translate the proteins, whereas the RNA viral polymerase generally binds to the 3′ end of the same RNA molecule to initiate replication. Thus, it is necessary to identify a balance between these two viral events [79, 80]. This balance or “switch” is performed through ribonucleoprotein complexes (RNPs) located in subcellular membranes, as described above. The flavivirus RNA viral genome contains two UTRs that have various functions, such as initiating and regulating viral translation, as well as viral complex replication and assembly at membrane fractions, through interactions with host cellular factors and nonstructural proteins that form RNPs (revised in [81]).

Most of these factors have been identified in mammalian cells, including BHK21 (for baby hamster kidney), Vero (green monkey kidney), HEK293A (human embryonic kidney-293), K562 (human erythroleukaemia cells), and U937 (a human monocytic cell line derived from a patient with generalized histiocytic lymphoma) cells. These cellular factors have been identified as elongation factor 1*α* (EF1*α*), Polypyrimidine Tract Binding (PTB) protein, the autoantigen-La (La protein), calreticulin, and nuclear factor 90 (NF90) [82–85]. However, little information is available about the discovery of the cellular factors in mosquito cells, such as C6/36 cells, compared with mammalian cells. Notably, EF1*α*, the La protein, eukaryotic initiation factor 5 (eIF5), 40S ribosomal protein S6, and 60S ribosomal protein L4 (Table 4) have been implicated in viral replication in mosquitoes [72, 83, 86, 87].

The cellular factors mentioned above, such as EF1*α* (highly conserved between different host species as mammals, chicken, and mosquitoes), translation initiation factor eIF5, and ribosomal proteins S6 and L4, participate in several steps of the translation process [88–91], except autoantigen-La, a nuclear protein involved in RNA polymerase III transcription termination [92, 93] and small RNA biogenesis, which acts as a chaperone and contributes to the retention of nascent RNA in the nucleus or stabilizes the RNA structure. The relocalization of this protein to the cytoplasm has been observed in several RNA viral infections, including flavivirus infections [94].

Additionally, eIF5 functions in cell proliferation, cell viability, and cell-cycle progression [95], and it is essential for cell survival [96]. A recent study reported that eIF5 possesses a higher similarity and shorter evolutionary distance in insects than in other organisms, suggesting that this protein plays an important and common physiological role. The ribosomal protein S6 (rpS6) is a component of the 40S ribosomal subunit, while the ribosomal protein L4 (RPL4 or RPL1) is a component of the 60S ribosomal subunit. While the precise function of rpS6 is currently under investigation, studies have shown that this protein is involved in regulating cell size, cell proliferation, and glucose homeostasis through the selective translation of particular classes of mRNAs. Both ribosomal proteins were discovered using tandem affinity purification assays in mosquito cells infected with WNV and DENV [72].

EF1*α* is involved in WNV and DENV replication and could act as a chaperone targeting the RNA to the viral replication compartments, as this protein is associated with the ER membrane fraction where RNA viral replication complexes are assembled [86, 88]. In infected mammalian cells, EF1*α* was colocalized with the WNV and DENV NS3 and NS5 (the viral RNA-dependent RNA polymerase-RdRP) proteins. This evidence suggests that EF1*α* is important for minus-strand RNA synthesis through interactions with the viral RNA and the replication complex proteins, including NS3 and NS5 [90, 97], and these interactions might be similar in mosquito cells.

Furthermore, the Shih group results suggested that eIF5 is upregulated in DENV-2-infected C6/36 cells, and this upregulated expression might play a role in preventing mosquito cell death in response to the viral infection. Thus, eIF5 facilitates continued viral growth and potentially persistent infection in mosquito cells, without affecting viral replication. This finding is supported by results in C6/36 cells treated with ciclopirox olamine (CPO), an eIF5 inhibitor, which did not affect the viral titres [87]. These results provide evidence that eIF5 plays a role in the interactions between the viruses and components of the host cells.

However, the interaction between the ribosomal proteins S6 and L4 and the NS2A and NS4B nonstructural viral proteins is likely involved in flavivirus RNA translation [72]. This report provided the first evidence of the binding of flaviviral proteins to either 40S or 60S ribosomal proteins. However, this interaction has been reported in other viruses, such as Epstein–Barr virus noncoding RNA EBER1, which recruits ribosomal protein L22 during infection, presumably, to aid in viral replication, or the hepatitis C (HCV) IRES region, which associates with the ribosomal S5 protein to position the HCV RNA on the 40S ribosomal subunit during translation [72]. The requirement of these translation factors during the viral genome processing or viral replication suggests that flaviviral proteins might have evolved mechanisms to bind and localize the proteins in appropriate compartments of the cell.

Moreover, the human La protein (47 kDa) in mosquito cells has a molecular weight of 50 kDa and potentially binds to the SINV RNA [98]. Because the La protein might function as an RNA chaperone, this protein could facilitate RNP assembly. In flaviviruses, such as JEV, the human La protein interacts with the viral 3′ SL RNA, and siRNA-mediated downregulation represses JEV replication [99]. In addition, in DENV, the human La protein interacts with two viral nonstructural proteins that form the DENV replicase complex: NS3 and NS5 [84]. However, the mosquito La protein also binds to the 3′ UTR of positive and negative polarity DENV RNAs and relocates to the cytoplasm in C6/36-infected cells [83]. These findings suggest a role for the human La protein in flaviviral replication as a component of the RNP. For example, DENV can be grown in human, primate, and mosquito cells but first requires viral growth in mosquitoes. Therefore, characterizing the cellular and viral proteins required for DENV translation and replication in mosquito cells is essential to understand the replicative cycle of the virus.

After analyzing this information, we proposed that the association of EF1*α* and the La protein, which function as chaperones, with eIF5 and ribosomal proteins S6 and L4, components of the translational machinery, could stimulate flavivirus translation in a favourable context. DENV infections induce the drastic rearrangement of the ER membranes, resulting in complex membranous structures that promote the switch from translation to viral replication once viral and/or cellular factors facilitate communication of the UTRs, thereby maximizing translation and replication as global processes. In addition to stabilizing RNA, the formation of viral ribonucleoprotein complexes (vRNPs) between the host cell factors and/or the viral proteins, as described above, likely regulates various steps of the viral life cycle. The formation of vRNPs might establish the genomic architecture and facilitate various processes in the viral life cycle, and the fact that flaviviruses share several cellular factors for translation/replication might explain the homologous viral interference frequently observed in both co- and superinfections in mosquitoes and mosquito-derived cell lines.

## 6. RNA Interference

The RNA interference (RNAi) response is one of the most important antiviral mechanisms in insects [100]. This response includes three main pathways to generate small RNAs that regulate gene expression: small interfering RNA (siRNA), microRNA (miRNA), and PiwiRNA [101]. The siRNAs are induced through the presence of double-stranded RNA (e.g., as a result of replicative intermediates or secondary structure of genome of RNA viruses), which is processed through the RNAse III-like enzymes Dicer-1 or Dicer-2 (primarily Dicer-2), to generate small dsRNA (21 to 25 nt). With the assistance of the R2D2 protein, these small RNAs are loaded into the RNA-induced silencing complex (RISC) involving the Argonaute proteins (Ago-1 or Ago-2). The synthesized siRNAs perfectly compliment the target RNA, resulting in RNA degradation through the exosome or the exoribonuclease XRN1/Pacman [100, 102].

The participation of the siRNA response in the regulation of flavivirus infection has been studied in both mosquito cells lines [74, 103–107],* Aedes aegypti* [104],* Culex pipiens quinquefasciatus* [108], and even in* Drosophila melanogaster* [103]. Several studies have indicated that the siRNA response is activated during DENV [74, 104, 106, 107] or WNV [103, 105, 108] infections, and the susceptibility to these flaviviruses increases when components of the siRNA pathway are silenced [103, 104, 106]. Moreover, C6/36 cells, which have a deficiency in the activity of the Dicer-2 enzyme, are more susceptible to DENV infection than Dicer-2 competent cells [74, 105, 107], and the natural polymorphism of the* Dicer-2* gene has been associated with the susceptibility of* Aedes aegypti* mosquitoes to some isolates of DENV-1 [109]. These findings strongly indicate the importance of siRNAs in the control of flaviviral infection.

Although mosquitoes have a competent RNAi pathway to degrade viral RNA, flaviviruses have mechanisms to evade the RNAi response and establish a persistent and noncytopathic infection in the vector [102]. One of these mechanisms might be the sequestration of the viral replicative complex into endoplasmic reticulum-derived vesicles; thus, the double-stranded viral RNAs generated during the viral replication cannot be sensed through Dicer-2 [73, 103, 104, 110]. Additionally, flaviviruses mutate at high rates, reflecting the absence of “proofreading” activity or RNA polymerases. Because the RNAi response is highly sequence-specific, these mutations in the viral genome might be another mechanism to evade this response [108].

However, viruses could express RNAi inhibitors. For example, mosquito and mammal cells infected with flaviviruses generate subgenomic RNA corresponding to the 3′ UTR of the viral genome through the participation of the cellular exoribonuclease XRN1, and this subgenomic RNA binds to Dicer-2 and Dicer-1, inhibiting the activity of these enzymes* in vitro* [1, 102]. In the case of DENV, the NS4B protein has an inhibitory effect in the RNAi response [111].

Moreover, the DIs in the siRNA pathway could be responsible for the homologous/heterotypic viral interference and persistent infection.* Drosophila melanogaster* cells persistently infected with several nonflavivirus RNA viruses generated cDNAs from the genomes of DIs through cellular LTR-retrotransposon reverse transcriptase-mediated retrotranscription. These cDNAs are apparently transcribed through the cellular transcription machinery to generate small double-stranded RNAs via several mechanisms, which are the source of the Dicer-2-RISC-produced interfering RNAs (RNAi) [21, 28] that control viral replication (Figure 1). Interesting similar results were obtained with the Sindbis virus, a member of Togaviridae, which shares similarities in the replicative cycle and genome type with members of the Flaviviridae family [28]. This finding suggests that a similar process might occur with flaviviruses; however, this idea requires further investigation. Furthermore, several retrotransposons with retrotranscriptase activity have been detected in* Aedes aegypti* [112–114],* Aedes albopictus*, and* Aedes polynesiensis* [112], suggesting that the same mechanism could operate in mosquitoes infected with flaviviruses; however, this hypothesis requires further investigation.

## 7. The Immune Response in Mosquitoes

The immune response in the mosquito might determine the susceptibility of these insects to arboviruses [115–117] and could be implicated in both the viral interference phenomenon and persistence. The immune response in mosquitoes is primarily regulated through three signalling pathways: immune deficiency (IMD), which mediates the production of antimicrobial peptides with activity against Gram-negative bacteria; the Toll-mediated pathway, which is involved in the defence against viruses, Gram-positive bacteria, and fungi and also stimulates the secretion of some antimicrobial peptides; and the Janus kinase-signal transducer and activator of transcription (JAK-STAT) pathway, which has been strongly associated with the antiviral response in* Aedes* mosquitoes [118–120].

Among the three main pathways involved in the immune response in mosquitoes, the Toll [14, 15, 72, 115, 116, 120] and JAK-STAT [15, 72, 115, 116, 119] pathways are apparently more important for controlling flavivirus infection. However, recent studies have suggested that the IMD response could play a secondary role [15, 120].

The participation of the immune response during flavivirus infection has primarily been studied with DENV [14–16, 115, 116, 119–121] in* Aedes aegypti* mosquitoes [14–16, 115, 116, 119], and this response has recently been characterized in the Aag2* Aedes aegypti* cell line [120]. However, the transcriptome analysis of* Aedes aegypti* mosquitoes infected with DENV, YFV, or WNV showed similar overall gene expression, indicating a conserved transcriptome signature [72].

During infections with DENV and other mosquito-borne flaviviruses, the first virus-vector interaction occurs in the midgut, where the immune response, known as the local immune response [121], is initially activated [15, 115, 119]. However, the systemic immune response represented by the abdominal fat body has also been implicated [121]. More recently, it has been reported that DENV upregulates the expression of salivary gland genes in* Aedes aegypti *mosquitoes that encode for proteins involved in the immune response and also induces the expression of a putative antibacterial, cecropin-like peptide, which exhibits activity against the four DENV serotypes and CHIKV [16].

Although the participation of the mosquito immune system has been clearly demonstrated during single flavivirus infection, there are no studies concerning the participation of this response in viral interference during co- or superinfections. Moreover, it is not clear whether immune system activation through a primary virus blocks the infection of a secondary virus. However, there is some indirect evidence suggesting the participation of the immune response in the viral interference phenomenon.* Culex quinquefasciatus* mosquitoes sequentially infected with WNV and SLEV through an oral route displayed lowered susceptibility to infection and lower dissemination rates of the second virus, but similar infection rates to the first virus. Some mosquitoes become infected with two viruses, but only one virus escapes to the midgut, suggesting the participation of the midgut infection barrier during secondary infection. Because WNV and SLEV belong to the same antigenic complex and both viruses are primarily transmitted through* Culex *spp., the primary virus likely blocks the infection of the secondary virus through the induction of the host antiviral response [36]. However, studies using the mosquito bacterial microflora have suggested that prior immune system activation influences the course of viral infection. The eradication of the endogenous bacterial flora in the midgut of* Aedes aegypti* mosquitoes treated with an antibiotic increased the viral titres in the mosquitoes infected with DENV-2 compared with a nontreated group. This effect is coincident with the ability of the bacterial flora to activate the Toll pathway and produce several antimicrobial peptides, such as cecropin, defensin, attacin, and gramicidin, in the midgut [15, 121]. A similar result was obtained with mosquitoes previously fed with either blood or sugar meals contaminated with* Proteus *sp. and* Paenibacillus *sp. [121]. Apparently, immune system activation through the microflora is not only limited to the midgut but also present in the abdominal fat body [121]. Interestingly, DENV-2 apparently reduces the microbial load in the midgut through the secretion of lysozyme C and cecropin G, indicating an interrelationship between the virus- and bacteria-induced immune responses [121]. A similar mechanism might operate during co- and superinfection with different flaviviruses and in homologous or heterotypic viral interference. However, as previously discussed, equilibrium between the viral replication and the antiviral response exists during persistent infection in mosquito cells [26, 27], and the precise participation of the immune response in persistent infection in mosquito cells and the mechanism used by the flaviviruses to circumvent this response remain unknown.

Although 80% of C6/36 cells persistently infected with JEV were positive for the NS5 protein based on immunofluorescence analysis [25], there is typically little correlation between the number of cells positive for the viral antigen and the release of infectious virus particles [122]. This finding has been reported in C6/36 cells persistently infected with DENV [22] and in different mosquito cells lines persistently infected with SLEV [61], reflecting the inhibition of virus particle production [22, 123] or the fact that initially all cells in culture support virus replication, but some cells stop replicating the virus and become resistant to superinfection during the late stages of infection. Eventually, the cells regain sensitivity to reinfection by the virus in the medium or the residual viral RNA in the cell, and thus the culture remains persistently infected [35]. However, another explanation is that a soluble antiviral factor is secreted from persistently infected cells. Pretreatment of TRA-171 cells with the supernatant from the same cells persistently infected with DENV reduces the viral titres of the four DENV serotypes [32]. Similarly, C6/36 cells pretreated with filtered culture medium from the same cells persistently infected with the four serotypes of DENV become resistant to reinfection with the same DENV viruses (heterotypic viral interference) but remain susceptible to infection with CHIKV. This effect is eliminated when the culture medium is preheated at 56°C for 30 minutes, suggesting the presence of a soluble, thermolabile, anti-DENV-specific factor [31]. Consistent with these findings, interferon and interferon-like substances are involved in heterologous interference [35], and Vago, a peptide with antiviral activity, has recently been identified and shown to be secreted from WNV-infected* Culex* mosquito cells,* Culex pipiens* mosquitoes, and DENV-infected RML12 cells (from* Aedes albopictus*) [124, 125]. Vago induction is Dicer-2-dependent, but RNAi-independent through the activation of TRAF, which cleaves the Rel2 protein (a homologue of mammalian NF*κ*B) and facilitates the translocation of this protein to the nucleus to activate the Vago gene [125]. However, the antiviral activity of Vago is mediated through the JAK-STAT pathway [124]. Although Vago expression is decreased in* Culex pipiens* mosquitoes at 8 days postinfection with WNV [124], the participation of Vago in viral interference during persistent infection cannot be excluded and will require further investigation.

## 8. Concluding Remarks

Flaviviral infection of the vectors is long-lived or persistent, and the mechanisms that participate in the establishment of this type of infection remain unknown. The present data suggest that this type of infection is a multifactorial phenomenon involving factors from the virus, such as defective interfering particles/genomes, and the host, such as the immune response, RNA interference, and cellular factors. The characterization of the mechanisms that participate in viral persistence is important to obtain a better understanding of the complex interactions between flaviviruses and mosquito cells to develop new strategies for control.

Viral interference is associated with, but not exclusive to, persistent infections. The cocirculation of two or more flaviviruses/alphaviruses in the same geographic area increases the opportunity for interviral interactions, and in most cases, the interaction between two mosquito-borne flaviviruses results in homologous viral interference, indicating that these viruses share mechanisms that regulate replication and several cellular factors required for viral translation/replication, a common requirement for different flaviviruses, which favours the conditions for competition.

The studies of viral interference in cell lines typically correspond to the results observed in mosquitoes, but the relevance of these findings to the dynamics of viral infection and transmission in nature requires additional studies. The viral interference experiments have primarily been performed with the* Aedes albopictus* mosquito cell line C6/36, a traditional cell line used as a model to study flavivirus infection in mosquitoes. However, recent studies have shown that this cell line contains a defective RNAi pathway, reflecting a defect in the Dicer-2 protein [74, 105]. Although the RNAi pathway is not the only factor that might participate in maintaining a persistent infection or explaining viral interference, these data indicate that these types of experiments should be performed using other cell lines, such as CCL-125, U4.4, and C7-10 cells, and vectors, such as* Aedes* and* Culex* mosquitoes. Moreover, these cell lines should be used to characterize the cellular factors involved in flavivirus replication and the pathways involved in the immune response to provide a cellular model to study the relevance of these phenomena in the transmission and epidemiology of mosquito-borne flaviviruses.

## Figures and Tables

**Table 1:** Mosquito cell lines persistently infected with flaviviruses.

Cell line	Insect	Virus	References
C6/36	*Aedes albopictus*	DENV-1–4	[31]

C6/36	*Aedes albopictus*	DENV-2	[22–24]

C6/36	*Aedes albopictus*	JEV	[25]

C6/36	*Aedes albopictus*	DENV-2 JEV Aal DNV	[44]

TRA-171	*Toxorhynchites amboinensis*	DENV-1–4	[32]

Sf9	*Spodoptera frugiperda*	SLEV	[49]

Ad1, Ad2 Ct1, Ct2 Aa1, Aa2	*Aedes dorsalis* *Culex tarsalis* *Aedes albopictus*	SLEV	[61]

DENV, Dengue virus; JEV, Japanese encephalitis virus; Aal DNV, *Aedes albopictus* densovirus; SLEV, Saint Louis encephalitis virus.

**Table 2:** Superinfections with flaviviruses in mosquito cell lines.

Cell line	Primary virus	Secondary virus	Type of interference	References
C6/36	DENV-1, DENV-2, DENV-3, or DENV-4	DENV-1, DENV-2, DENV-3, or DENV-4	Heterotypic	[31]

C6/36	DENV-1, DENV-2, DENV-3, or DENV-4 sylvatic and endemic strains	DENV-1, DENV-2, DENV-3, or DENV-4 sylvatic and endemic strains	Heterotypic	[43]

C6/36	DENV-2	DENV-2	None	[42]

C6/36	DENV-2	DENV-4	Heterotypic	[42]

C6/36	DENV-4	DENV-4	None	[42]

C6/36	DENV-4	DENV-2	None	[42]

C6/36	PCV	WNV or MVEV	Homologous	[12]

C6/36	CxFV	WNV	None	[45]

C6/36	NHUV	WNV or JEV or SLEV	Homologous	[48]

C6/36	DENV-2 and Aal DNV	JEV	None	[44]

C6/36	CxFV	WNV	Homologous	[47]

C6/36	DENV-1	DENV-3	Heterotypic	[30]

C6/36	DENV-3	DENV-1	Heterotypic	[30]

C6/36	DENV-1, DENV-2, DENV-3, or DENV-4	CHIKV	None	[31]

C6/36	DENV-3	CHIKV	None	[46]

C6/36	DENV-2	Aal DNV	None	[23]

C6/36	SINV	YFV	None	[35]

Sf9	SLEV	Baculovirus	None	[49]

C7-10	SINV	YFV	None	[35]

U4.4	SINV	YFV	None	[35]

*Ae. albopictus* cells	SINV	WNV	None	[29]

C6/36	DENV-4 or SINV	DENV-4 or SINV	Heterologous	[41]

C6/36	Aal DNV	DENV-2	Heterologous	[39]

TRA-171	DENV	VSV	Heterologous	[32]

*Ae. albopictus*, *Aedes albopictus*; DENV, Dengue virus; PCV, Palm Creek virus; WNV, West Nile virus; MVEV, Murray Valley encephalitis virus; JEV, Japanese encephalitis virus; SLEV, Saint Louis encephalitis virus; CHIKV, Chikungunya virus; CxFV, *Culex* flavivirus; NHUV, Nhumirim virus; Aal DNV, *Aedes albopictus* densovirus; SINV, Sindbis virus; YFV, Yellow fever virus; VSV, vesicular stomatitis virus.

**Table 3:** Coinfections with flaviviruses in mosquito cell lines.

Cell line	Viruses	Type of interference	References
C6/36	DENV-1 and DENV-3	Heterotypic	[30]

C6/36	DENV-2 and DENV-3	Heterotypic	[50]

C6/36	DENV-1, DENV-2, DENV-3, or DENV-4 (sylvatic and endemic strains)	Heterotypic	[43]

C6/36	DENV-2 and DENV-4	Heterotypic	[42]

Aag2	DENV-2 (clades NI-1 and NI-2B)	Heterotypic	[51]

C6/36	NHUV and WNV or SLEV or JEV	Homologous	[48]

C6/36	DENV-4 and SINV	Heterologous	[41]

C6/36	DENV-3 and CHIKV	Heterologous	[46]

DENV, Dengue virus; WNV, West Nile virus; JEV, Japanese encephalitis virus; SLEV, Saint Louis encephalitis virus; CHIKV, Chikungunya virus; NHUV, Nhumirim virus; SINV, Sindbis virus.

**Table 4:** Cellular factors involved in the flavivirus translation/replication.

Viruses	Mosquito host cell factors	Processes involved	References
DENV	EF1*α*, La	Replication	[83, 86]
	eIF5A	Infection	[87]
	40S ribosomal protein S6	Translation	[72]
	60S ribosomal protein L4		[72]

WNV	EF1*α*	Replication	[88]
	40S ribosomal protein S6	Translation	[72]

DENV, Dengue virus; WNV, West Nile virus.
